# Responsive Quaternized PDMAEMA Copolymers with Antimicrobial Action

**DOI:** 10.3390/polym13183051

**Published:** 2021-09-09

**Authors:** Theodore Manouras, Varvara Platania, Anthie Georgopoulou, Maria Chatzinikolaidou, Maria Vamvakaki

**Affiliations:** 1 Institute of Electronic Structure and Laser, Foundation for Research and Technology—Hellas, 700 13 Heraklion, Greece; mchatzin@materials.uoc.gr (M.C.); vamvakak@iesl.forth.gr (M.V.); 2 Department of Materials Science and Technology, University of Crete, 700 13 Heraklion, Greece; plataniavarvara@yahoo.com (V.P.); anthieg87@yahoo.com (A.G.)

**Keywords:** PDMAEMA, quaternization, antimicrobial polymers, *E. coli*, *S. aureus*, minimum inhibitory concentration, MIC, minimum bactericidal concentration, MBC

## Abstract

In this work, the antimicrobial action of partially quaternized poly(2-(dimethylamino)ethyl methacrylate) (PQDMAEMA) copolymers using different alkyl halides is presented. The poly(2-(dimethylamino)ethyl methacrylate) (PDMAEMA) homopolymer was synthesized by group transfer polymerization, followed by the modification of its tertiary amine groups, using bromoethane, iodoethane, bromohexane and bromoethanol, to introduce permanent cationic, quaternary ammonium salt moieties, randomly distributed along the polymer chains. In all cases, the degree of quaternization was low, at ~10 mol%, as verified by proton nuclear magnetic resonance spectroscopy to preserve the thermo-responsive character of the PDMAEMA precursor polymer. The biocidal activity of the lightly quaternized PQDMAEMA copolymers against *Escherichia coli* and *Staphylococcus aureus* was evaluated by calculating the minimum inhibitory concentration (MIC) as well as the minimum bactericidal concentration (MBC) of the polymers and by comparing them to the respective values of the precursor non-quaternized PDMAEMA homopolymer. The antibacterial mechanism of action in the solution was studied by zeta potential measurements, scanning electron microscopy and protein leakage tests signifying the disruption of the outer membrane of the bacterial cells to release their periplasmic proteins.

## 1. Introduction

Microbial infections and diseases induced by pathogens, such as, bacteria, viruses and fungi, comprise a major threat to human health [1]. These microorganisms can be transferred through air, water and food, thus complicating the prevention of their spreading and growth [2]. The development of antimicrobial compounds, including antibiotics, disinfectants and antiseptics, has been extensively employed to prevent microbial contamination [3]. However, the mutation of the microorganisms towards antimicrobial resistant bacterial strains, as a result of the extensive and injudicious use of antibiotics and disinfectants, has led to a necessity to develop new approaches to overcome the increasing risk of microbial infections [4,5].

To circumvent the microbial resistance to antibiotics, several broad-spectrum antimicrobial materials have been developed, which aim to kill the microorganisms either by photogenerated active species [6] or via biocidal moieties present in the material [7]. The killing mechanisms rely on the disruption of the outer membrane, the inhibition of the oxidative enzymes and interference with the DNA or RNA replication of the microorganism, leading to its death [8]. Photocatalytic TiO_2_ and ZnO nanoparticles have been employed to kill bacteria following UV irradiation. These nanoparticles can easily penetrate the cell wall, entering inside the bacteria and producing hydroxyl radicals upon light irradiation, which cause oxidative stress and damage to the bacteria lipids, proteins, and DNA [9,10]. Lately, the nanoparticles have been properly modified to enable their photocatalytic activation in the visible light regime, thus expanding their areas of application. Metallic nanoparticles, such as Ag and Cu, with dimensions ranging from 1 to 100 nm, have been also employed to inhibit bacterial growth and eventually induce cell death [11,12]. The antimicrobial action of these nanoparticles is based on four different mechanisms, operating simultaneously, and includes the adhesion of the nanoparticles onto the cell membrane, the penetration of the nanoparticles inside the bacteria to induce damage of the intracellular parts, the oxidative stress caused by the production of reactive oxygen species and the modulation of the signal transduction pathways of the bacteria [13]. Another class of effective antimicrobial materials are those based on cationic compounds in the form of small molecules or polymers and these have been widely used as platforms to address bacterial contamination both in solution and on surfaces. Among them, quaternary ammonium salts exhibit excellent biocidal activity against both gram-positive and gram-negative bacterial stains [14]. Antimicrobial surfaces have been prepared by either employing a synthetic quaternary ammonium salt-based polymer or by modifying natural polymers to introduce permanent cationic quaternary ammonium salt moieties [15,16]. Moreover, these polymers have been shown to act as effective biocides in bacteria dispersions. Their mechanism of action is based on the destabilization of the cell membrane, via the interaction of the cationic groups of the polymer with the negatively charged phospholipidic outer membrane of the bacteria, resulting in its disruption and rapid cell lysis. Similarly, random copolymers bearing protonated 4-ammoniumbuthylstyrene units have been employed as broad spectrum antibiotics with enhanced killing efficiency against both gram-positive and gram-negative bacteria strains [17].

Poly(2-(dimethylamino ethyl)methacrylate) (PDMAEMA) is a well-known pH- and temperature-responsive polymer. Its weak base tertiary amine groups become positively charged upon protonation below the effective p*K*_α_ of the polymer at ~pH 7, whereas they are neutral at higher pH values [18]. PDMAEMA can be also quaternized using different alkyl halides, which introduces permanent cationic quaternary ammonium salt moieties along the polymer chain. The low critical solution temperature (LCST) of the polymer is at ~40 °C and can be tuned by varying the degree of protonation or the degree of quaternization of its tertiary amine groups, as well as the alkyl chain length of the alkylating agent [19]. Quaternized PDMAEMA has been extensively used as an effective biocidal polymer against gram-positive and gram-negative bacterial strains, both in solution and on surfaces [20,21]. However, the need to fully quaternise the 2-(dimethylamino)ethyl methacrylate (DMAEMA) units, in order to ensure an effective biocidal action, remains unclear in the literature. In the present work, we demonstrate that even a small number of positively charged units along the polymer chains is sufficient to prevent bacterial growth in solution and therefore, lightly quaternized poly(2-(dimethylamino)ethyl methacrylate) (PQDMAEMA) copolymers, with degrees of quaternization as low as 10 mol%, can effectively kill both gram-positive and gram-negative bacterial strains. These copolymers retain their thermo-responsive character enabling the development of responsive antimicrobial polymers.

In this study, we report the synthesis, characterization and antimicrobial evaluation of PQDMAEMA copolymers using different alkylating agents. The precursor polymer was synthesized by group transfer polymerization, followed by the partial quaternization of its tertiary amine groups using iodoethane, bromoethane, bromohexane and bromoethanol. The PQDMAEMA copolymers preserve the thermo-responsive character of the PDMAEMA precursor and exhibit cloud points at higher temperatures. The degrees of quaternization of the random copolymers were determined by ^1^H NMR spectroscopy. The biocidal activity of the polymers in solution was evaluated in the growth of a gram-negative bacterium *Escherichia coli (E. coli)* and a gram-positive strain *Staphylococcus aureus (S. aureus)* via their minimum inhibitory concentration (MIC) and minimum bactericidal concentration (MBC) values. The bacterial morphology in the presence of the copolymers was visualized by scanning electron microscopy (SEM), while the protein leakage and zeta potential values of the bacteria cultures were measured to elucidate the antimicrobial mechanism.

## 2. Materials and Methods

### 2.1. Materials

All solvents and reagents were of analytical or HPLC grade and were purchased from Sigma-Aldrich (St. Louis, MO, USA) or Alfa Aesar (Thermofisher, Kandel, GmbH Germany). Tetrahydrofuran was refluxed at least three times over potassium metal and was freshly distilled before use. Methyl trimethylsilyl dimethylketene acetal was distilled under vacuum and was stored under nitrogen until use. The 2-(dimethylamino)ethyl methacrylate (DMAEMA) was passed through a basic alumina column to remove the inhibitor and any protic impurities and was double distilled over calcium hydride and 2,2-diphenyl-1-picrylhydrazyl before use. The tetrabutylammonium bibenzoate catalyst was prepared using the method of Dicker et al. [22], by the addition of two equivalents of benzoic acid to tetrabutylammonium hydroxide, and the product was recovered by filtration.

#### 2.1.1. Polymer Synthesis

All glassware were dried for three days at 160 °C, and were allowed to cool to room temperature in a desiccator before use. Syringes and stainless-steel needles were used to transfer the liquid reagents into the reaction vials. The polymerization reactions were carried out in 60 mL vials fitted with a rubber septum and a magnetic stirrer bar. In a typical synthetic procedure, first, the catalyst (1 mol% with respect to the initiator) was transferred to the reaction flask, followed by the addition of 15 mL dry THF. Next, the initiator (0.15 mL, 0.68 mmoles) was injected via a syringe followed by the addition of the monomer, DMAEMA (20 mL, 130 mmoles). The polymerization exotherm was monitored using a digital thermometer. When the temperature increase abated, the flask was opened to air and the polymer was obtained by precipitation in cold hexane and was dried overnight under reduced pressure. This was carried out as follows: ^1^H NMR (300 MHz, CDCl_3_, δ ppm): 0.8–1.1 (CCH_3_), 1.8 (CH_2_), 2.25 (N(CH_3_)_2_), 2.55 (CH_2_N) and 4.1 (OCH_2_).

#### 2.1.2. Quaternization Reaction

**Scheme 1:**
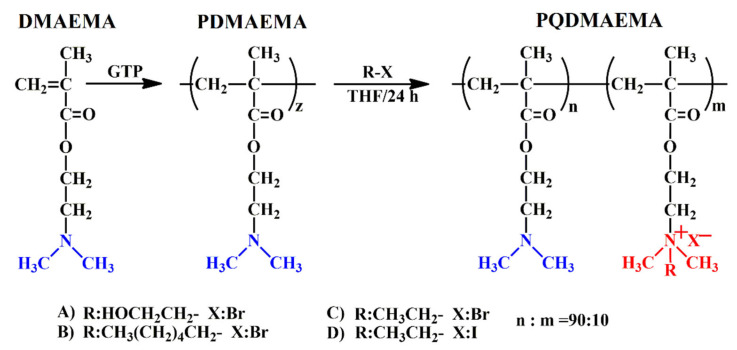
Synthesis of PDMAEMA homopolymer and quaternization reaction using different alkyl halides to obtain the PQDMAEMA copolymers.

The PDMAEMA homopolymer, synthesized by group transfer polymerization (GTP) as described above, was reacted with different alkyl halides, iodoethane, bromoethane, bromohexane and hydroxyethyl bromide, at RT to yield the PQDMAEMA copolymers (Scheme 1). The reaction was carried out in a round bottom flask using THF as the solvent. The theoretical degree of quaternization was set at 10 mol%, by adjusting the molar ratio of the alkyl halide to the DMAEMA monomer repeat units. The PQDMAEMA copolymers were isolated by precipitation in cold hexane and were characterized by ^1^H NMR spectroscopy to determine their actual degrees of quaternization.

### 2.2. Antimicrobial Evaluation

#### 2.2.1. Preparation of the Bacterial Suspension

The gram-negative *E. coli* strain DH10b and the gram-positive strain *S. aureus* were inoculated and cultivated in a Lysogeny broth (LB) and the broth was then incubated at 37 °C for 18–20 h at 200 r min^−1^. The concentration of bacterial suspension was then determined by measuring its optical density at 600 nm (OD600) using a spectrophotometer (Synergy HTX Multi-Mode Microplate Reader, BioTek, Winooski, VT, USA) and was diluted for further tests. Before each experiment, the optical density of the cell suspension was normalized to the optical density OD600 of 0.1, which corresponds to 2 × 10^7^ colony forming units (CFU) per ml through serial dilutions [23]. The bacterial cell number was then adjusted according to the protocols described above.

#### 2.2.2. MIC and MBC Tests

The MIC values were determined by the broth microdilution method. The original bacterial suspension is 2 × 10^6^ colony forming units (CFU) per mL in LB. The synthesized polymers were diluted in a phosphate buffer saline (PBS) solution at a concentration of 100 mg/mL and were tested at concentrations from 0.05 to 25 mg/mL. The diluted polymers were added to the bacterial suspensions to a total volume of 1 mL. The test tubes were then cultured at 37 °C for 24 h before visual observation. The minimum concentration of each sample whose solution remained clear after 24 h was determined as the MIC value. For the MBC test, 10 μL of the MIC and the above MIC concentrations were spread over the surface of agar plates and were incubated at 37 °C for another 24 h. The concentration in which the bacterial growth was 99.9% reduced compared to the control (bacterial suspension without the polymer) was recorded as the MBC. The results are presented as the mean of three independent experiments.

#### 2.2.3. Morphological Observation by SEM

The morphology of *E. coli* and *S. aureus* cultured in the presence of the polymer suspensions for 5 and 24 h was observed by SEM. The bacterial suspensions of *E. coli* and *S. aureus* was 2 × 10^7^ colony forming units (CFU) per mL, and were prepared according to a previously described protocol [24]. In detail, stock solutions of the polymers were prepared in PBS at a concentration of 10 mg/mL and were added to the bacterial suspensions up to a final concentration of 5 mg/mL. The samples were incubated at 37 °C, and after 5 and 24 h, 100 μL aliquots were placed on nitrocellulose (NC) filter membranes with a 0.2 μm pore size. After 5 min, the drop was dried, and another NC membrane was placed on top of the first one. All samples were then rinsed three times with PBS, fixed with 2 *v*/*v*% para-formaldehyde and 2 *v*/*v*% glutaraldehyde for 15 min and dehydrated in increasing concentrations (30–100 *v*/*v*%) of ethanol. The samples were finally dried using a critical point drier (Baltec CPD 030, BAL-TEC AG, Balzers, Liechtenstein, Switzerland), sputter-coated with a 20 nm thick layer of gold (Baltec SCD 050, BAL-TEC AG, Balzers, Liechtenstein, Switzerland) and were observed under a scanning electron microscope at an accelerating voltage of 15 kV (JEOL JSM-6390 LV, Tokyo, Japan).

#### 2.2.4. Kinetic Study

For the kinetic study, 50 μL of *E. coli* and *S. aureus* at a concentration of 2 × 10^5^ colony forming units (CFU) per mL were placed in each well in a 96 well plate. Stock solutions of the polymers in PBS were prepared, and 50 μL of each sample were added to the bacterial suspensions at final concentrations of 0.15, 0.3, 0.65, 1.25, 2.5 and 5 mg/mL. The bacterial suspensions in pure LB and in the presence of 100 μg/mL of the antibiotic amoxicillin/clavulanic acid were considered as the negative and positive controls, respectively. The 96 well plate was transferred to a spectrophotometer (Synergy HTX Multi-Mode Microplate Reader, BioTek, Winooski, VT, USA) and the absorbance was measured at 600 nm every 15 min for a total duration of 24 h. The results are presented as the mean of three independent experiments.

#### 2.2.5. Protein Leakage Test

For the protein leakage test, *E. coli* and *S. aureus* were harvested from 2.5 mL LB, washed twice with 1 mL PBS, and suspended in PBS until their concentration was 1 × 10^9^ colony forming units (CFU) per mL. Aliquots of 3.6 mL of the cell suspension were added to 0.4 mL of the diluted polymers (50 mg/mL) to obtain a final concentration of 5 mg/mL. An amount of 400 μL of the mixture was withdrawn immediately, filtered through a 0.2 μm membrane and set as the zero point. Then, 25 μL of the filtered sample were applied to the Coomassie Plus (Bradford) assay reagent according to the manufacturer’s instructions. The mixtures were incubated at 37 °C and 300 rpm for 1, 2, or 4 h, and 400 μL were then removed and filtered as described above. For all samples, the absorbance at 592 nm was measured and correlated to the protein concentration according to a standard curve. Bacteria cultured in pure LB in the absence of the polymers were used as the control. The experiments were carried out in triplicates.

#### 2.2.6. Statistical Analysis

Statistical analysis was performed for the protein concentration determination in the protein leakage assay using one-way ANOVA followed by Dunnett’s multiple comparisons test of the different polymers compared to the *E. coli* control at each experimental time point. Data were expressed as means ± standard deviation (SD). For this analysis the GraphPad Prism software version 8.0 (GraphPad Software, San Diego, CA, USA) was used. The symbol * designates statistically significant differences with *p* < 0.001.

### 2.3. Characterization Techniques

Size exclusion chromatography was carried out using a Waters system equipped with a Waters 515 HPLC pump, a Waters 2487 ultraviolet and a Waters 410 refractive index detectors (Waters, Milford, MA, USA). Two PLgel mixed D and mixed E columns (Agilent technologies, Santa Clara, CA, USA) at 40 °C were used for the size separation and the molecular weights and molecular weight distributions of the polymers were determined using five narrow PMMA standards, with molecular weights ranging from 850 to 342,900 g mol^−1^. Tetrahydrofuran (THF) was used as the eluent at a flow rate of 1 mL min^−1^. ^1^H NMR spectra were recorded on a Bruker Advance DPX 300 NMR Spectrometer (300 MHz) (Bruker, Billerica, MA, USA) using CDCl_3_ and D_2_O as the deuterated solvent for the PDMAEMA homopolymer and the PQDMAEMA copolymers, respectively. The polymer treated bacterial cultures were dried using a critical point drier (Baltec CPD 030, BALTEC AG, Balzers, Liechtenstein, Switzerland), sputter-coated with a 20 nm thick layer of gold (Baltec SCD 050, BAL-TEC AG, Balzers, Liechtenstein, Switzerland) and were observed under a scanning electron microscope at an accelerating voltage of 15 kV (JEOL JSM-6390 LV, Tokyo, Japan). Turbidimetry measurements were performed using a Perkin-Elmer LAMBDA 25 (Perkin Elmer corporation, Waltham, MA, USA) spectrophotometer equipped with a digital temperature controller. The optical density of 1 wt% aqueous solutions of the polymers at 650 nm, adjusted to pH 9.5, using 0.1 M NaOH, was followed as a function of the solution temperature, in the range between 25 and 95 °C at a heating rate of 1 °C min^−1^, to determine the cloud points of the polymers. The zeta potential values of the polymers and the *S. aureus* and *E. coli* cultures, with and without the polymers (5 mg/mL), were measured using a Malvern ZetaSizer, Nano ZS90 instrument (Malvern Panalytical, Malvern, Worcestershire, UK). Before the measurements, the bacteria were cultured overnight, washed three times with 0.5 mM PBS buffer (pH 7.2) at 3000 g and adjusted to an OD600 of 0.4. Zeta potential values were calculated from the electrophoretic mobility by the Smoluchowski equation at 25 °C.

## 3. Results and Discussion

**Figure 1:**
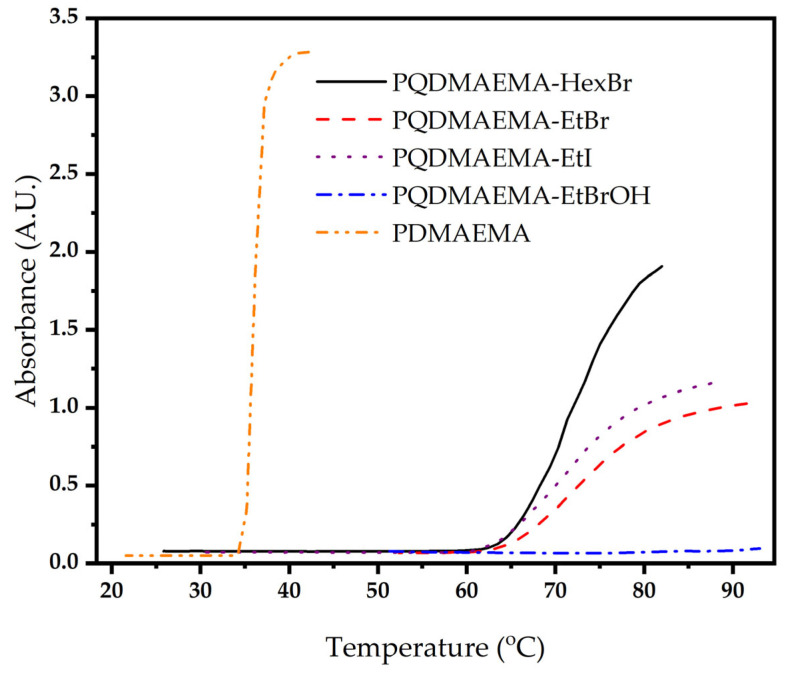
Absorbance vs. temperature curves for 1 wt% aqueous solutions of the PDMAEMA, PQDMAEMA-EtI, PQDMAEMA-EtBr, PQDMAEMA-HexBr and PQDMAEMA-EtBrOH copolymers at pH 9.5.

A well-defined PDMAEMA homopolymer with molecular weight *M*_n_ = 24,000 g mol^−1^ and molecular weight distribution *M*_w_/*M*_n_ = 1.1 was synthesized by group transfer polymerization. The homopolymer was modified via a facile quaternization reaction using different alkyl halides to introduce cationic quaternary ammonium salt moieties along the polymer chains. Four random copolymers, PQDMAEMA-EtBrOH, PQDMAEMA-EtI, PQDMAEMA-EtBr and PQDMAEMA-HexBr, were prepared using bromoethanol, iodoethane, bromoethane and bromohexane as the alkyl halides, respectively. The successful quaternization of the PDMAEMA homopolymer was verified by ^1^H NMR spectroscopy and the respective spectra are presented in Figures S1–S5. The degrees of quaternization of the PDMAEMA homopolymer were calculated by ratioing the integrals of the peaks which correspond to the methylene protons next to the nitrogen atom, before and after quaternization, at 2.6 ppm and 4.4 ppm, respectively. The degrees of quaternization calculated by ^1^H NMR spectroscopy were found to be 10.5 mol% for PQDMAEMA-EtI, PQDMAEMA-EtBr and PQDMAEMA-EtBrOH and 11.0% for PQDMAEMA-HexBr. The molecular weights of the quaternized polymers were calculated from the molecular weight of the PDMAEMA homopolymer and the respective alkylating agent, using the degree of quaternization of each copolymer found by ^1^H NMR spectroscopy, and were found to be 26,500, 25,700, 26,000 and 26,800 for PQDMAEMA-EtI, PQDMAEMA-EtBr, PQDMAEMA-EtBrOH and PQDMAEMA-HexBr, respectively. The temperature-responsive behavior of the copolymers in water was studied by turbidimetry measurements (Figure 1). PQDMAEMA-EtI, PQDMAEMA-EtBr and PQDMAEMA-HexBr retained their temperature responsive behavior, similar to the PDMAEMA homopolymer precursor, whereas, PQDMAEMA-EtBrOH did not exhibit a cloud point. An increase of the cloud point from 34 °C for the PDMAEMA homopolymer to 62 °C, 63 °C and 63 °C for the PQDMAEMA-EtI, PQDMAEMA-EtBr and PQDMAEMA-HexBr copolymers, respectively, was found, suggesting that the quaternization of the amine groups increases the hydrophilicity of the polymer and therefore its transition temperature. On the other hand, PQDMAEMA-EtBrOH was the most hydrophilic from the aforementioned copolymers, due to the presence of a hydroxy group in each monomer repeat unit, and therefore did not exhibit a cloud point.

The antibacterial action of the polymers, PQDMAEMA-EtBrOH, PQDMAEMA-EtI, PQDMAEMA-EtBr, PQDMAEMA-HexBr and PDMAEMA, was tested by means of the MIC and MBC tests. The results are shown in Table 1. The MIC and MBC values for the PDMAEMA homopolymer, and the PQDMAEMA-EtBrOH, PQDMAEMA-EtI, PQDMAEMA-EtBr and PQDMAEMA-HexBr copolymers were found to be between 0.2 to 2.4 mg/mL. Comparing the two bacteria strains, the MIC values were found to be lower against *E. coli* compared to *S. aureus*, while the MBC values were in a similar range of 0.4–1.6 mg/mL. Specifically, the MIC values against *E. coli* were found to be 0.2 mg/mL for all polymers except for PDMAEMA, which was 0.5 mg/mL, whereas the MIC values against *S. aureus* were found 0.4 mg/mL, except for PQDMAEMA-EtI, which was found 0.8 mg/mL and for PDMAEMA at 1 mg/mL.

The comparison of the antimicrobial values of the PQDMAEMA-EtBr and PQDMAEMA-EtBrOH copolymers suggests that the presence of a hydroxyl group in the quaternization agent, intended to render the quaternized polymer more hydrophilic, does not improve its antimicrobial efficacy. Finally, the lowest MIC and MBC values of 0.2 and 0.2 mg/mL, respectively against *E. coli*, and 0.4 and 0.8 mg/mL, respectively against *S. aureus* were found for the PQDMAEMA-HexBr polymer, which bears a longer alkyl chain length of six carbon atoms at each quaternary ammonium salt moiety. PDMAEMA quaternized with rosin have been evaluated against *E. coli* and *S. aureus* bacterial strains, showing 0.064–0.256 mg/mL MIC values depending on the degree of quaternization and the molecular weight of the copolymers [25]. In addition, 100% quaternized PDMAEMA with bromoethanol shown MIC values 0.5 mg/mL against S. aureus and 1 mg/mL against E.coli [26].

**Figure 2:**
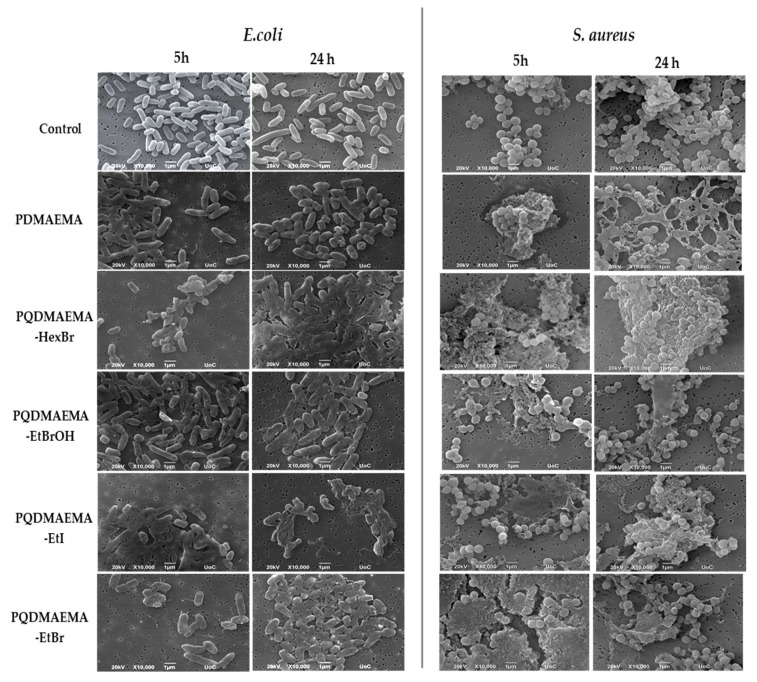
Representative scanning electron microscopy images of *E. coli* (**left**) and *S. aureus* (**right**) following incubation with 5 mg/mL cationic polymers for 5 and 24 h. The control represents the morphology of cells cultured in the absence of the polymers. Magnification is 10,000× and the scale bars represent 1 μm.

The morphological changes of the *E. coli* and *S. aureus* cell membranes, following incubation with 5 mg/mL cationic polymers for 5 and 24 h, were observed by SEM and were compared to the membranes of the non-treated bacteria. As shown in Figure 2 (left), after 5 h of incubation, the *E. coli* cells appeared with a flattened morphology and their cell membranes were damaged in contrast to the physiological rod-shaped bacteria (untreated control). Even more pronounced was the effect after 24 h of incubation, when the shrinkage of the bacterial membrane was dramatically increased, suggesting that severe cell damage had taken place. Extensive bacterial membrane damage was observed for the PQDMAEMA-EtI after 5 h and for PQDMAEMA-HexBr after 24 h. Similarly, representative SEM micrographs of *S. aureus* (Figure 2, right) showed that the bacterial cell surface of the untreated control group appeared smooth, intact with the typical characteristics of the native cells, while cells treated with all five polymers showed extensive cell membrane disruption, a fragmented morphology, leakage, and agglomerate formation after 5 h and 24 h of incubation.

**Figure 3:**
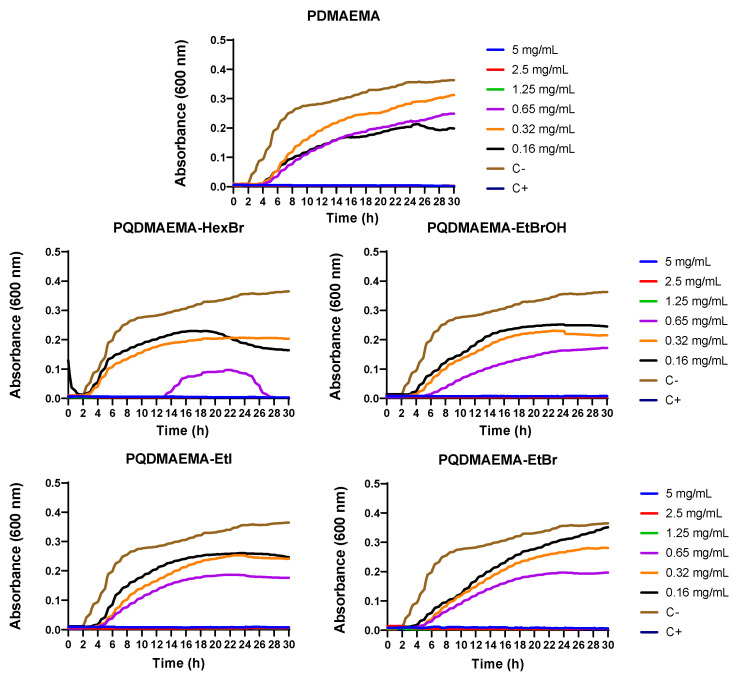
Growth kinetics of *E. coli* in the presence of polymer concentrations ranging from 0.16 to 5 mg/mL. The optical densities were measured for 30 h incubation at 37 °C using a multi-detection microplate reader at 600 nm and were automatically recorded for each well every 15 min. In all experiments, the bacterial suspension in pure LB and in the presence of amoxicillin/clavulanic acid were measured as the negative (C−) and positive (C+) control, respectively.

**Figure 4:**
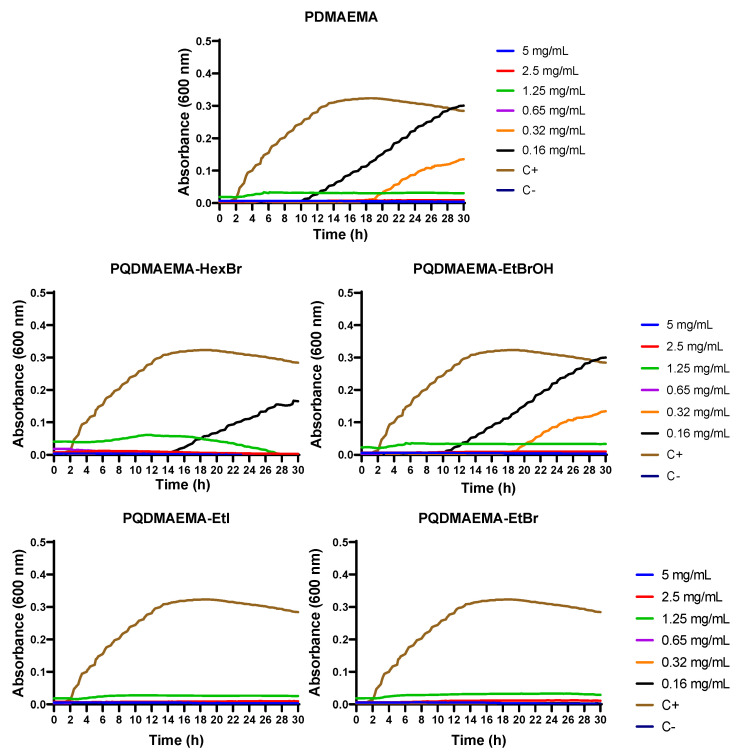
Growth kinetics of *S. aureus* in the presence of polymer concentrations ranging from 0.16 to 5 mg/mL. The optical densities were measured for 30 h incubation at 37 °C using a multi-detection microplate reader at 600 nm and were automatically recorded for each well every 15 min. In all experiments, the bacterial suspension in pure LB and in the presence of amoxicillin/clavulanic acid were measured as the negative (C−) and positive (C+) control, respectively.

The growth kinetics of *E. coli* and *S. aureus* were monitored in 100 μL LB, supplemented with the cationic polymers at different concentrations from 0.16 to 5 mg/mL, for 30 h incubation time and are presented in Figure 3 and Figure 4, respectively. For both bacterial strains, the increase in the polymer concentration resulted in an increase of the lag phase and a total reduction of the bacterial population within 30 h. All five polymers, even at the lower concentrations, were able to reduce the final bacterial population. This effect was higher for *E. coli* compared to *S. aureus*. This behavior may be due to the greater resistance of *S. aureus* against the PQDMAEMA copolymers. For *E. coli,* at PDMAEMA concentration higher than 1.25 mg/mL a bactericidal effect was detected, whereas, for *S. aureus* the corresponding concentration was 5 mg/mL. The PQDMAEMA-EtI copolymer exhibited the strongest antibacterial activity against *S. aureus* as evidenced from the inhibition of the growth at the concentration of 0.65 mg/mL.

**Figure 5:**
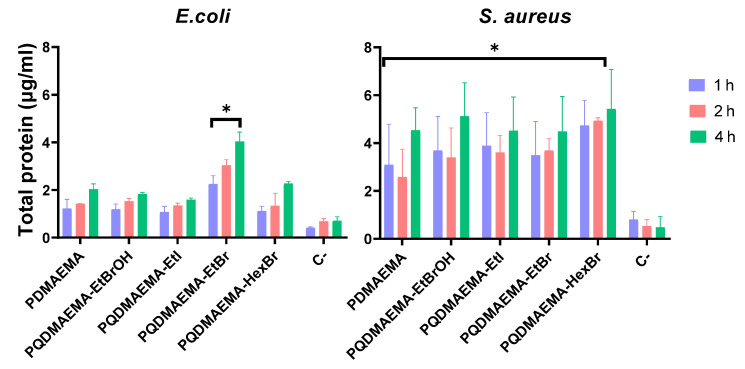
Protein leakage determined by the Bradford assay for *E. coli* and *S. aureus* incubated in the presence of the polymers at a concentration of 5 mg/mL, or in the absence of the polymers (negative control, C−) for 1, 2 or 4 h. The asterisk (*) designates significant differences in the presence of the different polymers compared to the *E. coli* and *S. aureus* control at each experimental time point (*p* < 0.001).

Finally, the leakage of the periplasmic proteins from the bacteria was investigated to further understand the action mode of the cationic polymers. The protein leakage from cells treated with all five polymers was higher than that found for the untreated cells of both strains (Figure 5). All the cationic polymers induced the leakage of the proteins from the first hour of incubation at 37 °C, and protein leakage increased after 4 h. This suggests that the quaternized polymers lead to the disruption of the outer membrane of the *E. coli* and *S. aureus* cells, resulting in the leakage of the periplasmic proteins, which is in agreement with the SEM images discussed above (Figure 2). In particular, the total amount of protein leaked from the *E. coli* cells, cultured in the presence of the cationic polymer PQDMAEMA-EtBr, was significantly higher compared to the untreated control. Protein leakage from *S. aureus* cells, treated with all five polymers, was significantly higher than that of the control cells. Notably, higher quantity of proteins were leaked through the *S. aureus* membranes than the *E. coli* membranes, suggesting that the sensitivity of the gram-positive *S. aureus* cells was higher than that of the gram-negative *E. coli,* cells, which is in good agreement with the results of the bacterial growth kinetics in the presence of the copolymers, discussed above (Figure 3 and Figure 4). Similar sensitivity of gram-positive bacteria compared to gram-negative ones has been reported in a previous study using self-assembled polypeptide nanogels [27]. However, the higher quantity of leaked proteins from the S. aureus cells compared to the E. coli cells did not translate into lower MIC values for the former cells. Similar results were reported by Alfei et al. who evaluated a 4-ammoniumbuthylstyrene-based random copolymer against several gram-positive and gram-negative bacterial strains. They also found that the MIC and MBC values against *E. coli* were lower compared to the respective values against S. aureus [17]. We attribute this behavior to the complex interactions between the cells and the biocidal species which we consider that are unique and characteristic for each biocide-cell pair. By comparing the findings from the MBC tests with the results from the protein leakage tests, one can conclude that even the PDMAEMA homopolymer can result in the disruption of the outer membrane of the bacteria to release the periplasmic proteins. However, this does not necessarily lead to bacteria death, as suggested by the SEM images of the bacteria (Figure 1), from which it was shown that the incubation with the PDMAEMA homopolymer caused significantly less damage to the cells compared to those incubated with PQDMAEMA-EtBr, PQDMAEMA-EtI, PQDMAEMA-HexBr and PQDMAEMA-EtBrOH.

**Figure 6:**
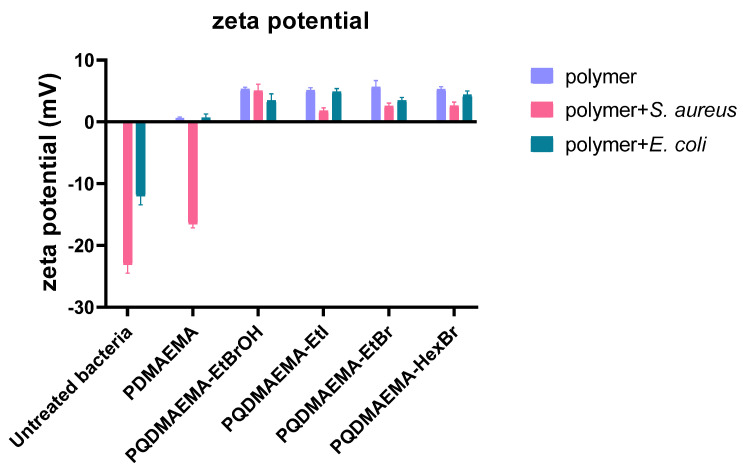
Zeta potential values for 5 mg/mL polymer solutions in LB (blue bars), and *E. coli* (green bars) and *S. aureus* (pink bars) suspensions incubated with 5 mg/mL cationic polymers in LB (blue bars).

To further elucidate the role of surface charge on the interaction of the bacteria with the cationic polymers, zeta potential measurements were carried out (Figure 6). The bacterial suspensions showed a negative surface charge, whereas, the quaternized polymers in the LB medium exhibited positive zeta potential values, as expected. However, following the addition of the polymers to the bacterial suspensions, the zeta potential values were all found to be positive and only slightly lower than those measured for the neat polymers, suggesting that the positively charged polymer chains interacted with the negatively charged outer membrane of the bacterial cells, causing a charge reversal. On the other hand, the bacterial suspensions incubated with the PDMAEMA homopolymer showed zero or negative zeta potential values, suggesting that the polymer was unable to cause charge reversal of the cell membrane. This was the case in particular for *S. aureus*, which was attributed to the low degree of protonation of the polymer in the medium. The above results verify the electrostatic interactions between the positively charged quaternized copolymers and the negatively charged cells, which lead to the shrinkage of the bacterial membrane and cause severe cell damage and bacterial killing as discussed above.

The cytotoxic effect of the PDMAEMA homopolymer and the PQDMAEMA copolymers on murine fibroblasts L929 was studied in vitro. Polymer dilutions were selected based on the MBC values determined above. The cell viability was quantified at concentrations of 0.4, 0.8, 1.6 and 3.2 mg/mL of the polymer for 24 and 48 h in culture. All five polymer samples were found to induce a significant decrease in cell proliferation, compared to the TCPS control, for all four polymer concentrations after 24 and 48 h in the culture (Figure S6).

## 4. Conclusions

Partially quaternized PDMAEMA copolymers were synthesized via the post polymerization modification of the tertiary amine groups of a PDMAEMA homopolymer using iodoethane, bromoethane, bromoethanol and bromohexane as the alkylating agents. The successful quaternization of the polymer was verified by ^1^H NMR spectroscopy and the degrees of quaternization were calculated at ~10 mol%. The PQDMAEMA-EtI, PQDMAEMA-EtBr and PQDMAEMA-HexBr copolymers retain the temperature responsive behavior of the precursor PDMAEMA homopolymer with clouds points at higher temperatures due to their more hydrophilic nature, which is attributed to the permanent cationic moieties of the quaternized DMAEMA units. PQDMAEMA-EtBrOH did not exhibit a cloud point in the temperature range measured suggesting that the hydroxyl group further increases the hydrophilicity of the copolymer. The quaternized polymers were evaluated against *E. coli* and *S. aureus* bacterial strains by calculating their MIC and MBC values and comparing them to the respective values found for the precursor PDMAEMA homopolymer. The quaternized polymers showed similar MIC values to the PDMAEMA homopolymer, ranging from 0.2–0.8 mg/mL for *E. coli*, and from 0.4–0.8 mg/mL for *S. aureus*. The MBC values were found to range from 0.4–3.2 mg/mL for *E. coli*, and from 0.8–1.6 mg/mL for *S. aureus*. PQDMAEMA-HexBr showed the lowest MIC and MBC values at 0.2 and 0.4 mg/mL, respectively for *E. coli*, and 0.4 and 0.8 mg/mL, respectively for *S. aureus*. The morphology of the bacteria, following incubation with the quaternized polymers, suggested a significant cell membrane disruption for both strains in the presence of the cationic polymers. The growth kinetics of *E. coli* and *S. aureus* showed that the polymers caused a delay in the growth of the bacterial cells between 2 h and 4 h even at lower polymer concentrations. The PQDMAEMA-HexBr polymer demonstrated the biggest delay of 10 h in *E. coli* growth at the concentration of 0.65 mg/mL, and this is in agreement with the MBC value of 0.4 mg/mL. The delay in growth was higher for *E. coli* compared to *S. aureus*, which may be attributed to the greater resistance of *S. aureus* against the PQDMAEMA copolymers. The PQDMAEMA-EtI polymer exhibited the strongest inhibition against *S. aureus* at 0.65 mg/mL. Furthermore, protein leakage measurements proved the disruption of the outer membrane of the bacteria, following incubation with the polymers. These findings suggest that the cationic polymers lead to an effective cell death. Finally, zeta potential measurements proved the mechanism of action of the cationic copolymers, which interacted electrostatically with the negatively charged cell membranes of both strains, leading to a charge reversal from negative values for the bacterial suspension in the absence of the polymers, to positive zeta potential values following incubation of the bacteria with the cationic copolymers.

## Figures and Tables

**Table 1:** MIC and MBC values of the cationic polymers prepared in this work and evaluated against *E. coli* and *S. aureus*.

Materials	*E. coli*		*S. aureus*	
	MIC (mg/mL)	MBC (mg/mL)	MIC (mg/mL)	MBC (mg/mL)
PDMAEMA	0.5	0.8	1.0	2.4
PQDMAEMA-EtBr	0.2	0.2	0.4	0.8
PQDMAEMA-EtI	0.2	1.6	0.8	1.6
PQDMAEMA-HexBr	0.2	0.2	0.4	0.8
PQDMAEMA-EtBrOH	0.2	0.2	0.4	1.6

## Data Availability

Data are available upon request.

## Supplementary Materials

The following are available online at www.mdpi.com/article/10.3390/polym13183051/s1, Figures S1–S5 show the ^1^H NMR spectra for PDMAEMA, PQDMAEMA-EtBr, PQDMAEMA-EtI, PQDMAEMA-HexBr and PQDMAEMA-EtBrOH, respectively, Figure S6 shows the cytotoxicity assessment of PDMAEMA and the PQDMAEMA copolymers and Figure S7 shows higher magnification SEM images of E. coli.
