# Supplementary material for: Responsive Quaternized PDMAEMA Copolymers with Antimicrobial Action
Source: Polymers (Basel). 2021 Sep 9;13(18):3051. doi: 10.3390/polym13183051 (PMC8472408; doi:10.3390/polym13183051)
Supplement: Supplementary file 1 [file polymers-13-03051-s001.zip › polymers-1371258 revised Supporting information (1).pdf]

polymers

MDPI

Supplementary Materials

Responsive quaternized PDMAEMA copolymers with antimicrobial action

Theodore Manouras,[1,2*] Varvara Platania,[2] Anthie Georgopoulou,[2] Maria Chatzinikolaidou[1,2] and Maria Vamvakaki[1,2]

1 Institute of Electronic Structure and Laser, Foundation for Research and Technology– Hellas, 700 13 Heraklion, Crete, Greece
2 Department of Materials Science and Technology, University of Crete, 700 13 Heraklion, Crete, Greece
* Correspondence: tman@iesl.forth.gr

Citation: Manouras, T.; Platania, V.; Georgopoulou, A.; Chatzinikolaidou, M.; Vamvakaki, M. Responsive Quaternized PDMAEMA Copolymers with Antimicrobial Action. *Polymers* **2021**, *13*, 3051. https://doi.org/10.3390/polym13183051

Academic Editors: Spiros H. Anastasiadis, Hermis Iatrou and Asterios (Stergios) Pispas

Received: 23 August 2021
Accepted: 8 September 2021
Published: 9 September 2021

Publisher's Note: MDPI stays neutral with regard to jurisdictional claims in published maps and institutional affiliations.

Copyright: © 2021 by the authors. Licensee MDPI, Basel, Switzerland. This article is an open access article distributed under the terms and conditions of the Creative Commons Attribution (CC BY) license (http://creativecommons.org/licenses/by/4.0/).

Materials and methods

Cell culture maintenance

L929 fibroblastic cells (DSMZ Braunschweig, Germany, ACC-2) established from normal subcutaneous areolar and adipose tissue of a male C3H/An mouse were used for the cell viability and proliferation assessment as a relevant cell type for the biocompatibility testing of biomaterials according to the ISO 10993-5 standards. Cells were cultured in RPMI 1640 medium supplemented with 10% fetal bovine serum (FBS), 100 units/ml of penicillin, 100 mg/ml of streptomycin and 1% amphotericin (all from Gibco). Cells were maintained in a humidified atmosphere under 5% CO2 at 37°C, and the media were changed twice a week.

Cell cytotoxicity assessment

Polymer cytotoxicity was evaluated and quantitatively assessed using the resazurin-based metabolic assay PrestoBlue® (Invitrogen, USA) as previously described [1]. For the assessment of the polymer samples, 104 cells per well were seeded in 96 well plates. After 24 h, the culture medium was replaced with medium containing the different dilutions of the polymer samples. Cell viability was measured at days 1 and 2. The measurements were performed in a spectrophotometer (Synergy HTX Multi-Mode Microplate Reader, BioTek, Bad Friedrichshall, Germany) and the absorbance was measured at 570 and 600 nm. Cells cultured on the tissue culture treated polystyrene (TCPS) surface was used as control. Data represent means ± standard deviation of triplicates of two independent experiments (n = 6).

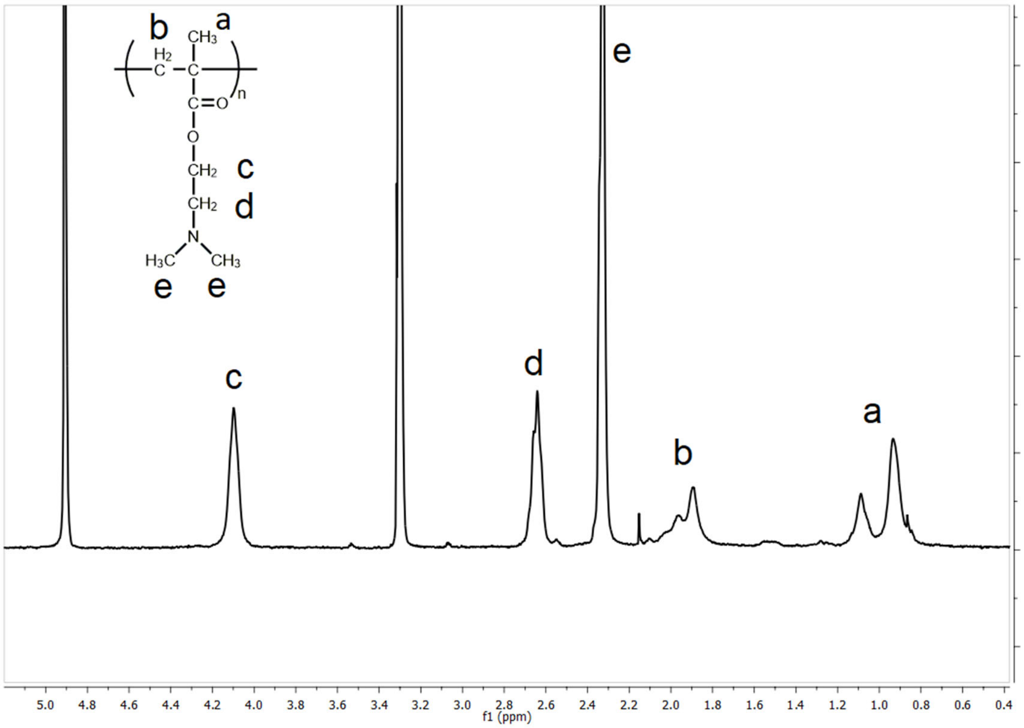

Figure S1: ^{1}H NMR spectrum of the PDMAEMA homopolymer in MeOD.

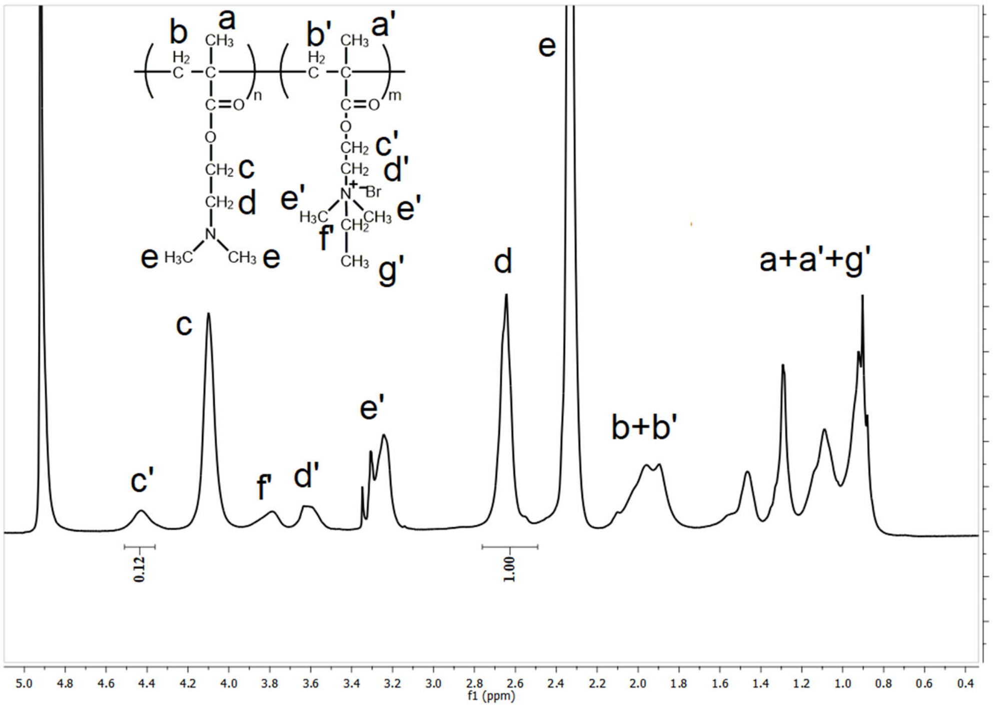

Figure S2: ^{1}H NMR spectrum of PQDMAEMA-EtBr in D_2O.

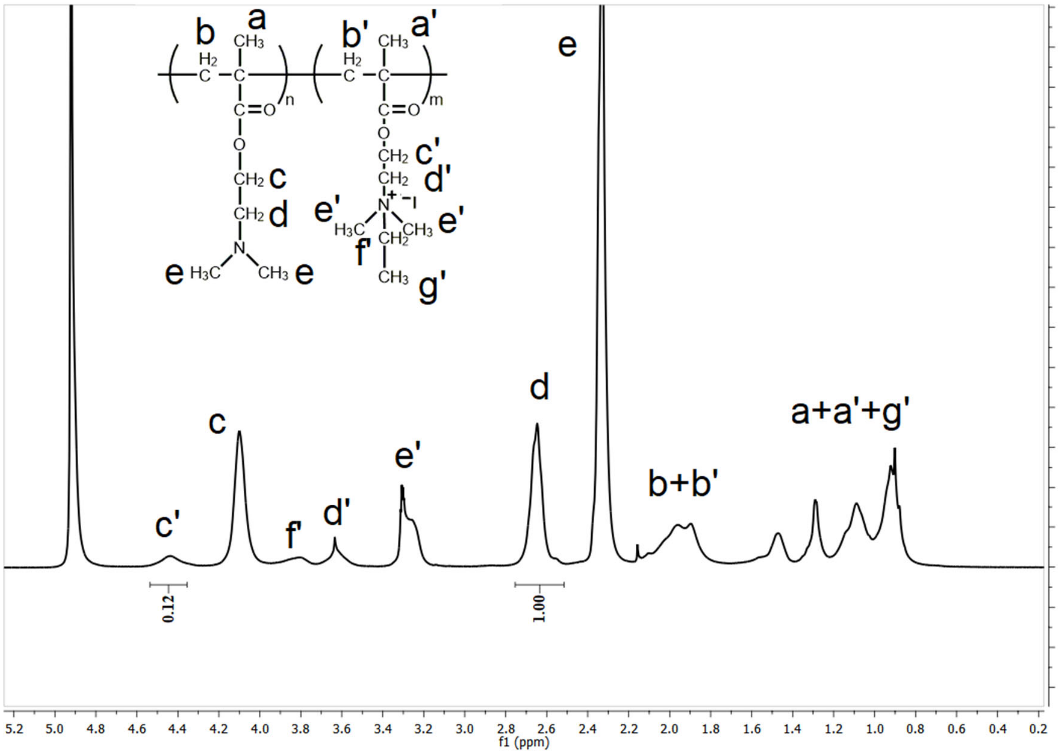

Figure S3: ^{1}H NMR spectrum of PQDMAEMA-EtI in D_2O.

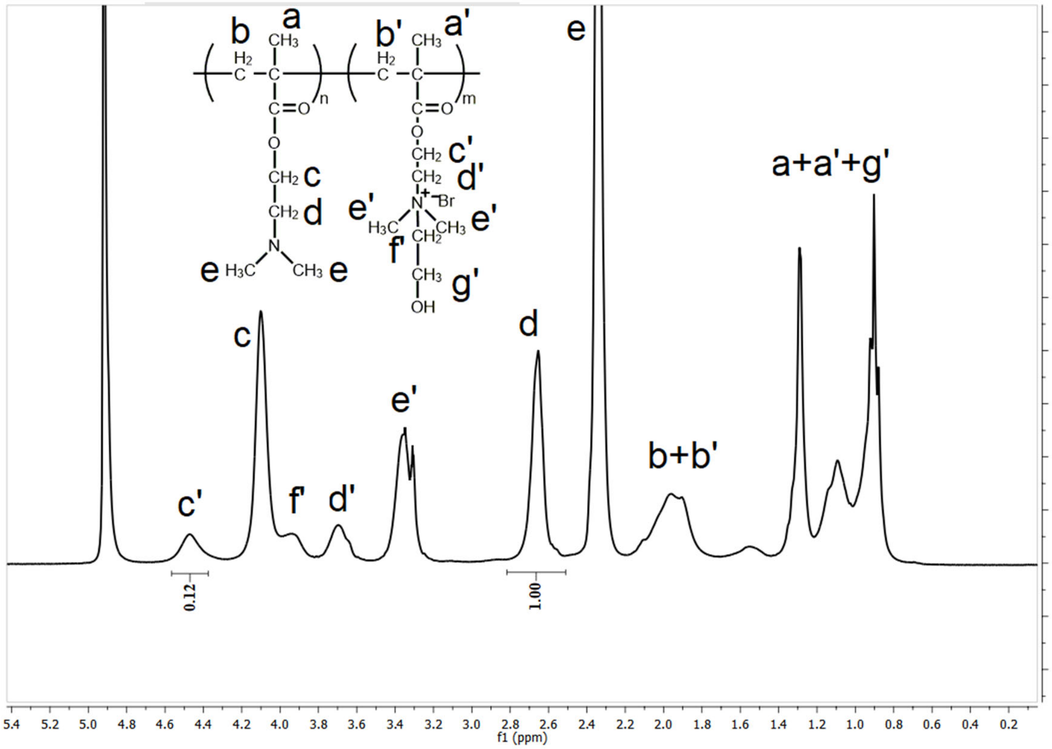

Figure S4: ^{1}H NMR spectrum of PQDMAEMA-EtBrOH in D_2O.

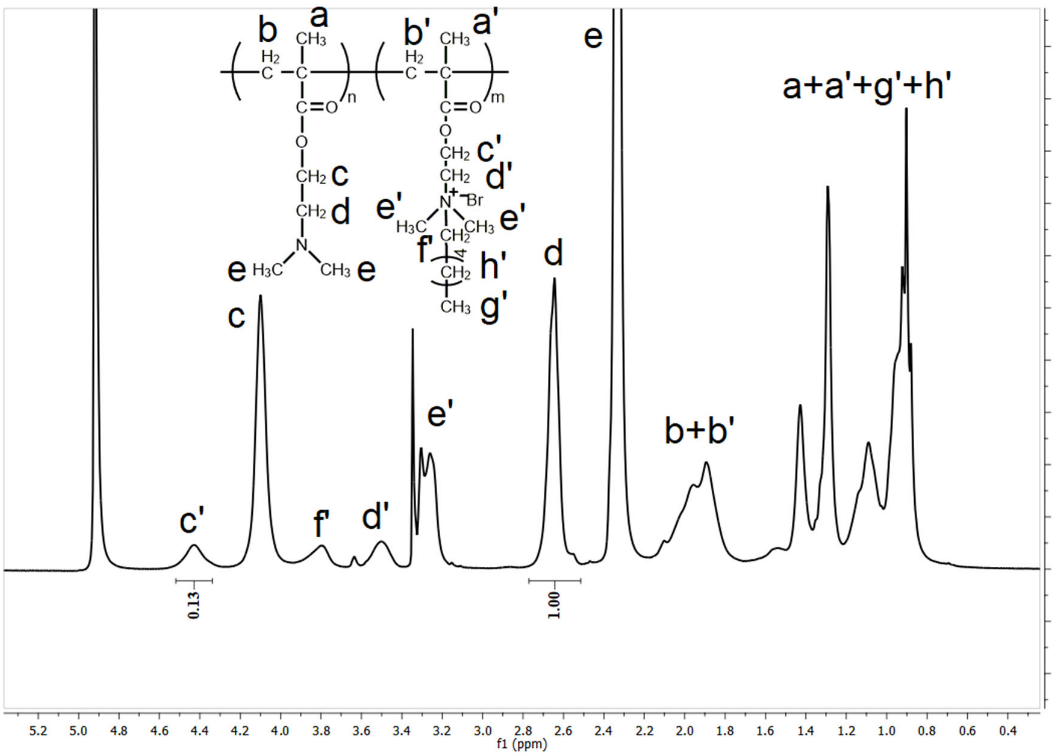

Figure S5: ^{1}H NMR spectrum of PQDMAEMA-HexBr in D_2O.

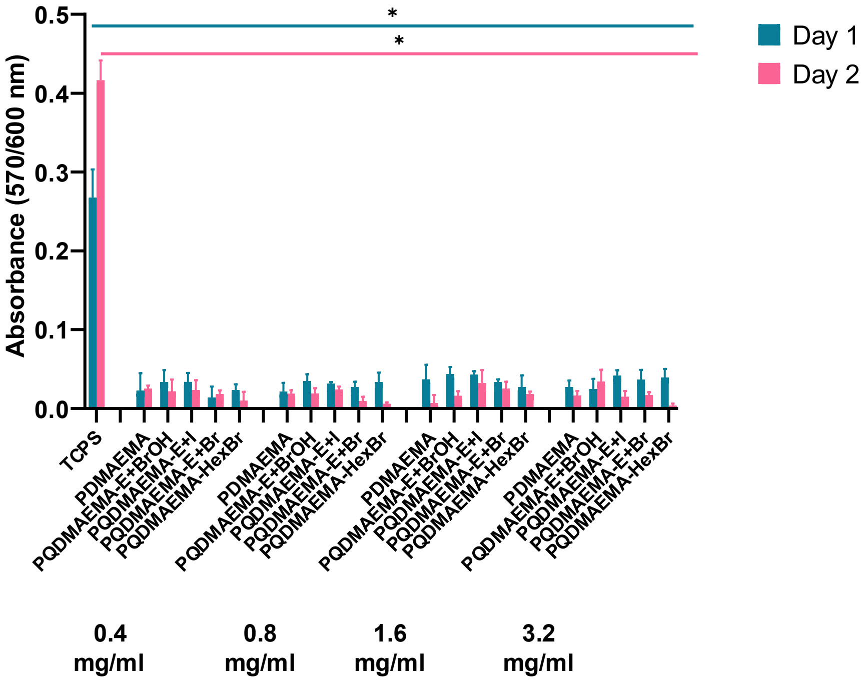

Figure S6. Cytotoxicity assessment of PDMAEMA and the PQDMAEMA copolymers at 0.4, 0.8, 1.6 and 3.2 mg/mL polymer concentration, using the resazurin-based cell viability reagent PrestoBlue®. The experimental data were analyzed using two-way ANOVA followed by the Tukey's multiple comparisons test between the five polymers at each

concentration group and the TCPS control. For these analyses the GraphPad Prism software version 8.0 (La Jolla, USA) was used. The values represent means ± standard deviation of triplicates of two independent experiments (n = 6), and the asterisk (*) designates significant differences compared to the TCPS control (p = 0.0001).

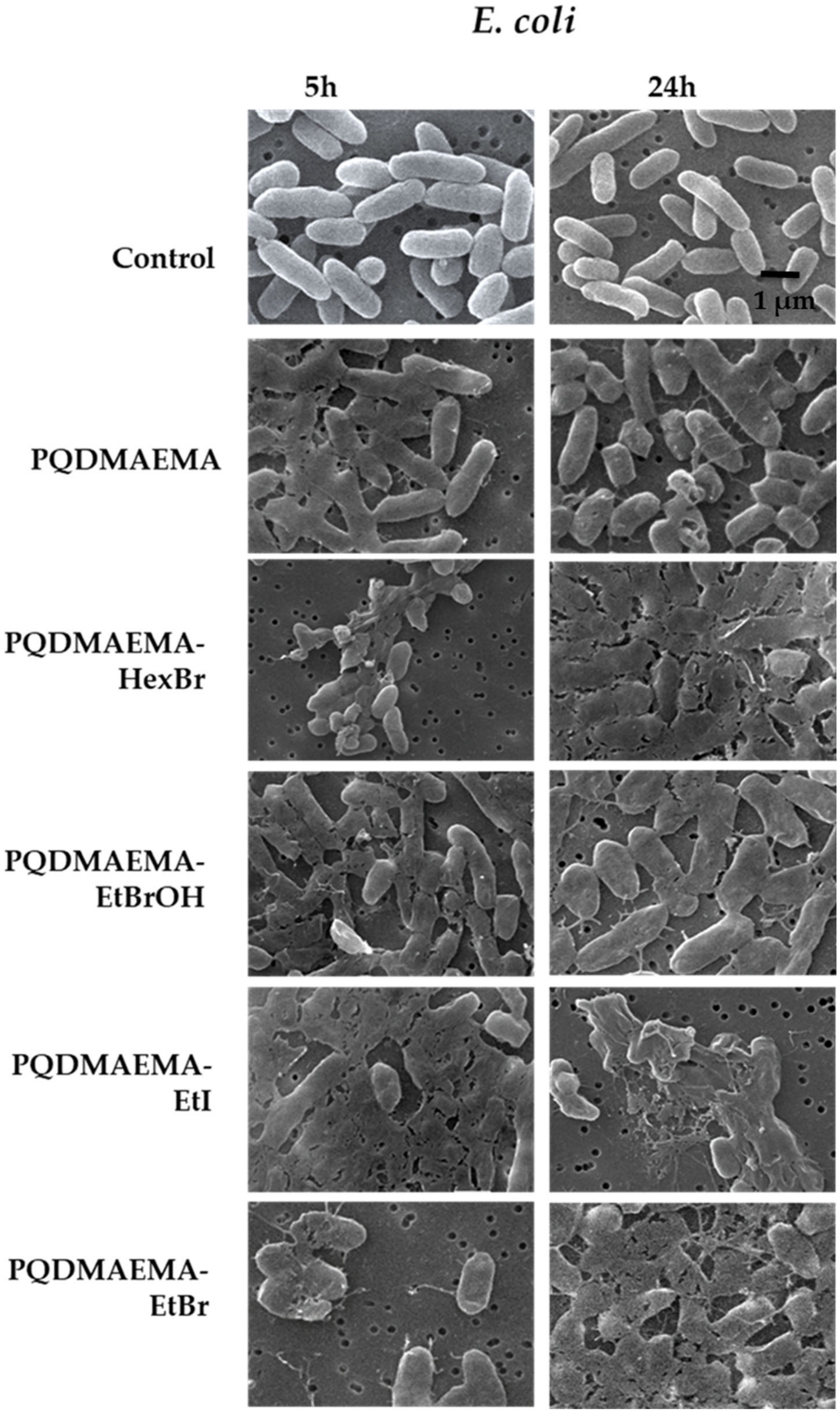

Figure S7. Zoomed-in SEM images of E. coli focusing on morphological changes on bacterial cell membranes after incubation with 5 mg/ml cationic polymers for 5 and 24 h. The control represents the morphology of cells cultured in the absence of the polymers.

References

1. Kavasi, R.M. and C.C. Coelho, *In Vitro Biocompatibility Assessment of Nano-Hydroxyapatite.* 2021. **11**(5).
